# Macrophage phagocytosis checkpoints in DLBCL immunotherapy: an evidence-maturity framework from CD47 to CD200

**DOI:** 10.3389/fonc.2026.1933140

**Published:** 2026-09-11

**Authors:** Shasha Qi, Lu Deng, Shan Luo, Yuhong Yao, Tao Chen

**Affiliations:** 1 Department of Hematology, The Second Affiliated Hospital of Guizhou University of Chinese Medicine, Guiyang, China; 2 The Affiliated Hospital of Guizhou Medical University, Guiyang, China

**Keywords:** antibody-dependent cellular phagocytosis, anti-CD20, CD200/CD200R, CD47, diffuse large B-cell lymphoma, immune checkpoint, tumor-associated macrophages

## Abstract

Diffuse large B-cell lymphoma (DLBCL) is treated with an expanding range of antibody- and cell-based therapies, yet macrophage phagocytosis checkpoints are still often discussed one molecule at a time. Here, we ask how far the evidence for each major axis has actually progressed in DLBCL immunotherapy. We considered five areas of support: lymphoma protein-level evidence, antibody-dependent cellular phagocytosis (ADCP), *in vivo* lymphoma data, clinical lymphoma evidence, and biomarker or predictive relevance. CD47/SIRPalpha remains the reference point because it has the broadest mechanistic, lymphoma, and clinical support. Major histocompatibility complex class I (MHC-I)/leukocyte immunoglobulin-like receptor B1 (LILRB1) and CD39/CD73-adenosine signaling have meaningful lymphoma-related functional evidence but remain less mature clinically. CD24/Siglec-10 is still best regarded as candidate/emerging. CD200/CD200R sits at a different stage: its myeloid biology, receptor structure, and Dok2-RasGAP signaling make it a credible candidate, but a direct DLBCL experiment showing restoration of anti-CD20 ADCP after pathway blockade is still lacking. We also consider how these pathways may be shaped by Fc receptor competence, spatial tumor-macrophage contact, anti-CD19 therapy, bispecific antibodies, and CAR-T-cell therapy. The purpose of the framework is practical: to separate what is already supported in lymphoma from what still needs to be tested before clinical claims are made.

## 1 Introduction

Diffuse large B-cell lymphoma (DLBCL) is the most common aggressive B-cell lymphoma, but it is not a single biological entity. Gene-expression studies divide conventional DLBCL into germinal center B-cell-like (GCB) and activated B-cell-like (ABC) cell-of-origin groups. In routine pathology, non-GCB is often used as a practical immunohistochemical surrogate for ABC biology, although the two are not identical. Primary mediastinal large B-cell lymphoma (PMBCL) is a distinct large B-cell lymphoma with characteristic molecular and immune features. These differences matter when immune mechanisms are discussed, because both tumor-cell programs and the surrounding microenvironment vary across these disease contexts (1–7).

Treatment has also changed substantially. Rituximab, cyclophosphamide, doxorubicin, vincristine, and prednisone (R-CHOP) remains an important first-line backbone, while polatuzumab vedotin plus rituximab, cyclophosphamide, doxorubicin, and prednisone (pola-R-CHP) is used for selected previously untreated patients in the United States and Europe. For patients with primary refractory disease or relapse within 12 months, CD19-directed chimeric antigen receptor T-cell (CAR-T) therapy has become an established option when patients are eligible. Later-line treatment may include CD20/CD3 bispecific antibodies, antibody-drug conjugates, and other targeted approaches. Tafasitamab is relevant here because it is an Fc-engineered anti-CD19 antibody and therefore extends the macrophage question beyond anti-CD20 therapy. In practice, treatment choice and sequencing still depend on prior therapy, fitness, access, transplant or CAR-T eligibility, and disease context (8–16).

Within this changing treatment landscape, tumor-associated macrophages (TAMs) are more than a measure of immune-cell abundance. They can participate directly in the clearance of antibody-opsonized lymphoma cells through fragment crystallizable (Fc) receptor-dependent mechanisms, including antibody-dependent cellular phagocytosis (ADCP). Whether that happens efficiently depends on the state of the macrophage, the balance of activating and inhibitory Fc receptors, physical access to the tumor cell, and the inhibitory signals present at the same time. These factors are unlikely to be identical from one DLBCL case to another or to remain unchanged during treatment (17–19).

This review compares the major macrophage phagocytosis checkpoints in that treatment context and places them at different levels of evidence maturity. CD47/signal regulatory protein alpha (SIRPalpha) is used as the benchmark. Major histocompatibility complex class I (MHC-I)/leukocyte immunoglobulin-like receptor B1 (LILRB1) and CD39/CD73-adenosine signaling are placed in an emerging tier, while CD24/sialic acid-binding Ig-like lectin 10 (Siglec-10) remains candidate/emerging. CD200/CD200R is kept at the validation frontier because the biological rationale is strong enough to justify focused testing, but the key DLBCL protein, spatial, signaling, and antibody-enhanced phagocytosis data are not yet in place.

## 2 Tumor-associated macrophages in DLBCL immunotherapy

Tumor-associated macrophages are common in the DLBCL microenvironment and can contribute to tumor-cell clearance, antigen presentation, cytokine production, stromal remodeling, and immune suppression. A high macrophage count by itself, however, says little about what those cells are doing. DLBCL tissues can contain macrophage populations with quite different functional states, and CD163-enriched populations have been associated with adverse outcome in several studies, although this relationship has not been uniform across cohorts or treatment settings (20–24).

The familiar M1/M2 terminology is convenient, but human DLBCL macrophages rarely fit neatly into either category. M1-like states are generally linked to inflammatory signaling, antigen presentation, and markers such as HLA-DR or CD80/CD86. M2-like or suppressive states more often show CD163, CD206, IL-10-associated, scavenger, or tissue-remodeling features. In lymphoma tissue, these programs can overlap within the same population, and treatment may shift macrophage states rather than simply switch them from M1 to M2 or vice versa. CD68, CD163, or CD206 staining should therefore not be taken as a direct readout of phagocytic competence (20–22).

For ADCP, Fc receptor composition is likely to be more informative than an M1/M2 label alone. Human macrophages can express the high-affinity Fc gamma receptor I (FcγRI/CD64), activating FcγRIIA (CD32A) and FcγRIIIA (CD16A), and the inhibitory FcγRIIB (CD32B). Their relative expression helps determine how strongly an IgG-opsonized target is recognized and engulfed (25–29). The same macrophages may carry SIRPalpha, LILRB1, CD200R, Siglec-10, adenosine receptors, and, in some states, programmed cell death protein 1 (PD-1). Receptor expression is context dependent. A macrophage can therefore be checkpoint-positive without being strongly Fc-competent, or Fc-competent without being close enough to the lymphoma cell to phagocytose it.

### 2.1 Conceptual temporal model of TAM remodeling during DLBCL immunotherapy

Before therapy, DLBCL tissues may contain heterogeneous TAM niches with variable Fc gamma receptor competence, suppressive-marker expression, and proximity to malignant B cells. Some tumors may contain CD163- or CD206-enriched macrophage neighborhoods with low baseline ADCP competence, whereas other tumors may be macrophage-rich but spatially or functionally disconnected from antibody-opsonized tumor cells.

Shortly after anti-CD20 exposure, Fc gamma receptor engagement may trigger a transient inflammatory and phagocytic phase. In this early treatment window, inflammatory monocyte or macrophage recruitment and tumor-cell engulfment may coexist with compensatory suppressive feedback, including induction of IDO, ARG1, PD-L1, CD39/CD73-adenosine signaling, or alternative macrophage programs. This dual response provides a plausible explanation for why an initially immune-activating antibody treatment can still generate a suppressive myeloid niche if negative feedback dominates.

At relapse or after repeated antibody exposure, macrophages may acquire a therapy-adapted suppressive phenotype characterized by altered chemokine responsiveness, checkpoint co-expression, metabolic suppression, and reduced ADCP capacity. CXCR4-, CD11b-, IDO-, ARG1-, PD-L1-, or adenosine-pathway-associated programs should be interpreted as validation candidates rather than fixed DLBCL relapse markers. This model is designed to guide longitudinal sampling rather than to claim a proven universal DLBCL trajectory.

This temporal view also helps avoid treating TAMs as a static cell population. A checkpoint that is weak before therapy may become more relevant after Fc receptor engagement, cytokine release, or repeated antibody exposure. Conversely, a ligand that is present at baseline may be functionally irrelevant if receptor-bearing macrophages are spatially separated from malignant B cells or lack phagocytic competence.

### 2.2 Spatial macrophage-checkpoint ecology in DLBCL

DLBCL should not be interpreted as a randomly mixed suspension of malignant B cells and immune cells. Spatially resolved transcriptomic studies have shown that macrophage states in DLBCL are spatially organized and clinically relevant. Digital spatial profiling of CD68-positive cells has generated spatially derived macrophage signatures that differ across reactive lymphoid tissue and DLBCL, associate with cell-of-origin subtypes, and stratify overall survival in multiple DLBCL cohorts (23). More recent multi-modal spatial transcriptomic and proteomic profiling of DLBCL has defined distinct cellular niches with different cellular compositions, spatial organizations, and patterns of intercellular communication (24).

Spatial organization is particularly important for membrane checkpoints. CD47/SIRPalpha, CD200/CD200R, CD24/Siglec-10, and MHC-I/LILRB1 can only signal when tumor cells and receptor-bearing macrophages are close enough to interact. CD39/CD73-adenosine is different because suppression can extend beyond direct cell contact through extracellular purine metabolism. For practical purposes, the tissue can therefore be viewed as containing contact-dependent checkpoint niches, broader paracrine or metabolic suppressive zones, and architectural compartments such as tertiary lymphoid structure (TLS)-like regions, perivascular areas, stromal regions, and tumor-rich neighborhoods that determine whether macrophages and lymphoma cells actually meet.

Spatial validation should therefore measure more than ligand positivity. It should quantify receptor-positive TAM density, tumor-macrophage distance, nearest-neighbor interactions, ligand-receptor co-expression within cellular neighborhoods, and whether checkpoint-positive tumor cells are physically accessible to Fc gamma receptor-competent macrophages. For CD200/CD200R, the key spatial question is whether CD200-positive lymphoma cells and CD200R-positive macrophages occupy the same contact-permissive niches. For CD39/CD73, the key question is whether adenosine-pathway activity overlaps with macrophage- and T-cell-rich regions in which paracrine suppression can plausibly operate.

## 3 Methods: hypothesis-oriented narrative review with structured evidence mapping

We conducted a hypothesis-oriented narrative review with structured evidence mapping. It was not designed as a meta-analysis or a protocol-driven systematic review. PubMed/MEDLINE and Web of Science Core Collection were the main structured sources. We supplemented them with targeted Google Scholar searches, citation tracking, selected lymphoma and cancer-immunology journal checks, and ClinicalTrials.gov. **Table 1**, **Supplementary Figure S1**, and **Supplementary Data Sheet 1** provide the working search record. Studies were prioritized when they reported lymphoma protein expression, macrophage phagocytosis or ADCP, antibody-based lymphoma models, *in vivo* lymphoma data, clinical development, or biomarker implications. Contemporary US and European DLBCL guidance and pivotal studies of anti-CD19 antibodies, antibody-drug conjugates, bispecific antibodies, and CAR-T therapy were checked separately to provide treatment context for Section 6 and **Table 2**. Those records were not used to raise or lower checkpoint evidence tiers.

**Table 1:** Search sources and working study-selection record.

Source	Search scope/terms	Records identified	Role in structured review
PubMed/MEDLINE	DLBCL/large B-cell lymphoma/B-cell lymphoma AND anti-CD20/ADCP/macrophage AND CD47, LILRB1, CD39/CD73, CD24, CD200, PD-1/PD-L1 or related terms.	252	Main peer-reviewed biomedical literature source.
Web of Science Core Collection	Five Topic-field searches covering DLBCL, macrophage-mediated phagocytosis, anti-CD20 ADCP, CD47/SIRPalpha, CD200/CD200R, and candidate myeloid/phagocytosis-checkpoint axes.	77; 133; 42; 213; 8	Structured database search performed to strengthen methodological reproducibility.
Google Scholar targeted hand-searching	Five targeted queries covering CD47/SIRPalpha, LILRB1, CD200/CD200R, CD24/Siglec-10, and CD39/CD73 in lymphoma phagocytosis contexts; first one to two result pages screened; citation chasing from anchor papers performed.	18 candidate records	Supplementary source for recent mechanistic papers and citation-chain checking.
ClinicalTrials.gov	magrolimab lymphoma rituximab; evorpacept lymphoma; CD47 lymphoma; SIRPalpha lymphoma.	42 records before de-duplication	Mapping of clinical development activity; not used as efficacy proof.
De-duplication and screening	Manual de-duplication across sources and title/abstract screening for lymphoma, macrophage, ADCP, and checkpoint relevance.	33 included in structured evidence mapping	Produced the structured evidence-mapping framework used for the present draft.
High-impact journal checking	Targeted checking of recent issues and article records from lymphoma, hematology, and cancer-immunology journals for macrophage checkpoint, anti-CD20, and ADCP terms.	Not counted separately	Risk-mitigation and update-checking step; no independent efficacy inference was drawn from this step.
Contemporary treatment-context checking	Current US and European LBCL guidance, together with targeted checks of anti-CD19 antibodies, antibody-drug conjugates, CD20/CD3 bispecific antibodies, and CAR-T therapy.	Not counted separately	Used to provide clinical context for Section 6 and **Table 2**. These records were not used to assign checkpoint evidence tiers.

**Table 2:** Modern DLBCL immunotherapy and macrophage checkpoint relevance.

Therapeutic context	Representative examples	Macrophage relevance	Checkpoint implication
Anti-CD20 antibodies	Rituximab, obinutuzumab	Direct Fc gamma receptor-mediated ADCP and macrophage effector engagement.	Most direct setting for testing CD47/SIRPalpha, LILRB1, CD39/CD73, CD24/Siglec-10, and CD200/CD200R blockade.
Fc-engineered anti-CD19 antibody	Tafasitamab plus lenalidomide	Tafasitamab can recruit Fc receptor-dependent effector functions. Preclinical lymphoma studies also show that CD47/SIRPalpha blockade can strengthen macrophage-mediated antitumor activity.	Provides an anti-CD19 setting for checkpoint testing, although the clinical tafasitamab-lenalidomide regimen does not isolate the macrophage contribution.
Antibody-drug conjugates	Polatuzumab vedotin, loncastuximab tesirine	Primary mechanism is payload delivery, but treatment-induced cell death may interact with antigen presentation and myeloid clearance.	Checkpoint profiling may inform combination or resistance biology but should not be overinterpreted.
CD20/CD3 bispecific antibodies	Glofitamab, epcoritamab, mosunetuzumab	Primary mechanism is T-cell redirection, but macrophages may shape antigen presentation, cytokine tone, and suppressive TME architecture.	Macrophage checkpoints may indirectly affect T-cell-engaging therapies through myeloid suppression and tissue-level immune state.
CAR-T-cell therapy	Axi-cel, tisa-cel, liso-cel	TAMs may influence cytokine networks, antigen presentation, inflammation, and T-cell persistence.	Candidate checkpoint profiling may be useful for combination hypotheses but requires mechanism-linked validation.
CD47/CD20 or SIRPalpha-CD20 constructs	Bispecific/fusion approaches	Designed to link B-cell targeting with macrophage checkpoint disruption.	Mechanistically attractive but clinical proof and safety differentiation remain incomplete.

Each checkpoint was judged in five areas: protein-level evidence in DLBCL or related large B-cell lymphoma (LBCL), functional evidence for antibody-dependent macrophage phagocytosis, *in vivo* lymphoma evidence, clinical lymphoma evidence, and biomarker or predictive relevance. **Table 3** summarizes the resulting tiers. An Established designation required direct lymphoma evidence together with substantial functional and/or clinical support. Emerging axes had at least one relevant primary lymphoma study, or strong mechanistic evidence with lymphoma-adjacent support, but incomplete independent or clinical validation. Candidate/Emerging was used when the biological case was meaningful but decisive DLBCL ADCP-rescue or clinical evidence was still missing. Candidate was reserved for indirect, non-lymphoma, review-level, or mainly hypothesis-generating evidence.

**Table 3:** Evidence-maturity map of macrophage phagocytosis checkpoints relevant to DLBCL immunotherapy.

Axis	Overall tier	Main supporting evidence	Main limitation/negative signal	Validation priority
CD47/SIRPalpha	Established benchmark	Rituximab-synergy and lymphoma phagocytosis models, *in vivo* lymphoma data, and multiple clinical lymphoma studies/trials (25–27, 31–34, 77).	Clinical development has not been uniformly successful; anemia/toxicity management, Fc design, mixed-cohort enrollment, terminated or inconclusive trials, and limited biomarker enrichment weaken direct maturity claims.	Refine biomarker-enriched CD47/SIRPalpha combinations and identify non-redundant co-checkpoints.
MHC-I/LILRB1	Emerging	Dual CD47/LILRB1 blockade enhanced CD20 antibody-dependent phagocytosis of lymphoma cells by macrophages, supporting a non-redundant co-checkpoint model (35, 36).	Clinical lymphoma evidence remains limited; paired tumor MHC-I/LILRB1 biology and ex vivo ADCP response require validation.	Test dual-axis blockade with CD20 antibodies and macrophage receptor profiling.
CD39/CD73-adenosine	Emerging	CD39 inhibition and adenosine-pathway targeting enhanced macrophage ADCP or anti-CD20 anti-lymphoma activity in B-cell lymphoma models (37, 38).	Mostly preclinical/translational; soluble metabolite effects may depend on tissue adenosine tone, macrophage state, and pharmacodynamic pathway inhibition.	Develop adenosine-pathway biomarkers and pharmacodynamic endpoints.
CD24/Siglec-10	Candidate/Emerging	Pan-cancer checkpoint biology plus mantle-cell lymphoma and aggressive LBCL immune-phenotype data (39, 40).	Direct DLBCL anti-CD20 ADCP restoration data are limited; lymphoma-adjacent signals should not be treated as DLBCL-definitive evidence.	Validate CD24-high, Siglec-10-positive macrophage-rich LBCL contexts.
CD200/CD200R	Validation-frontier Candidate/Emerging	CD200/CD200R is immunoregulatory; CD200 protein evidence is heterogeneous in LBCL with a stronger PMBCL context; recent phagocytosis-checkpoint biology, CD200-CD200R structure, and CD200R-Dok2-RasGAP signaling provide a coherent structural-functional rationale (48, 49, 52, 58–63).	No published DLBCL experiment yet shows that CD200/CD200R blockade restores rituximab- or obinutuzumab-mediated ADCP. This is the decisive translational gap linking protein expression, spatial proximity, signaling, and functional rescue.	Prioritize CD200-positive LBCL/DLBCL or PMBCL-like samples; test CD200 surface protein, CD200R-positive TAM proximity, signaling readouts, and anti-CD20 ADCP rescue after CD200/CD200R blockade.
Macrophage PD-1/PD-L1	Candidate/context	PD-1/PD-L1 checkpoint biology is established in lymphoma immunity, but macrophage-specific ADCP evidence in DLBCL remains limited (65, 66).	Evidence is less specific to anti-CD20 ADCP in DLBCL; best treated as immune-state context rather than a validated phagocytosis checkpoint.	Define whether macrophage PD-1/PD-L1 modifies ADCP or mainly reflects suppressive immune state.

Exploratory public transcriptomic observations were not allowed to change these evidence tiers. We used them only to help identify settings or samples that might be worth testing. Transcript abundance alone cannot show surface protein expression, receptor engagement, spatial proximity, macrophage suppression, treatment response, or rescue of ADCP. This limitation is especially relevant to CD200/CD200R and is illustrated in **Supplementary Figure S2**.

## 4 Antibody-mediated phagocytosis in anti-CD20 and anti-CD19 settings

**Figure 1:**
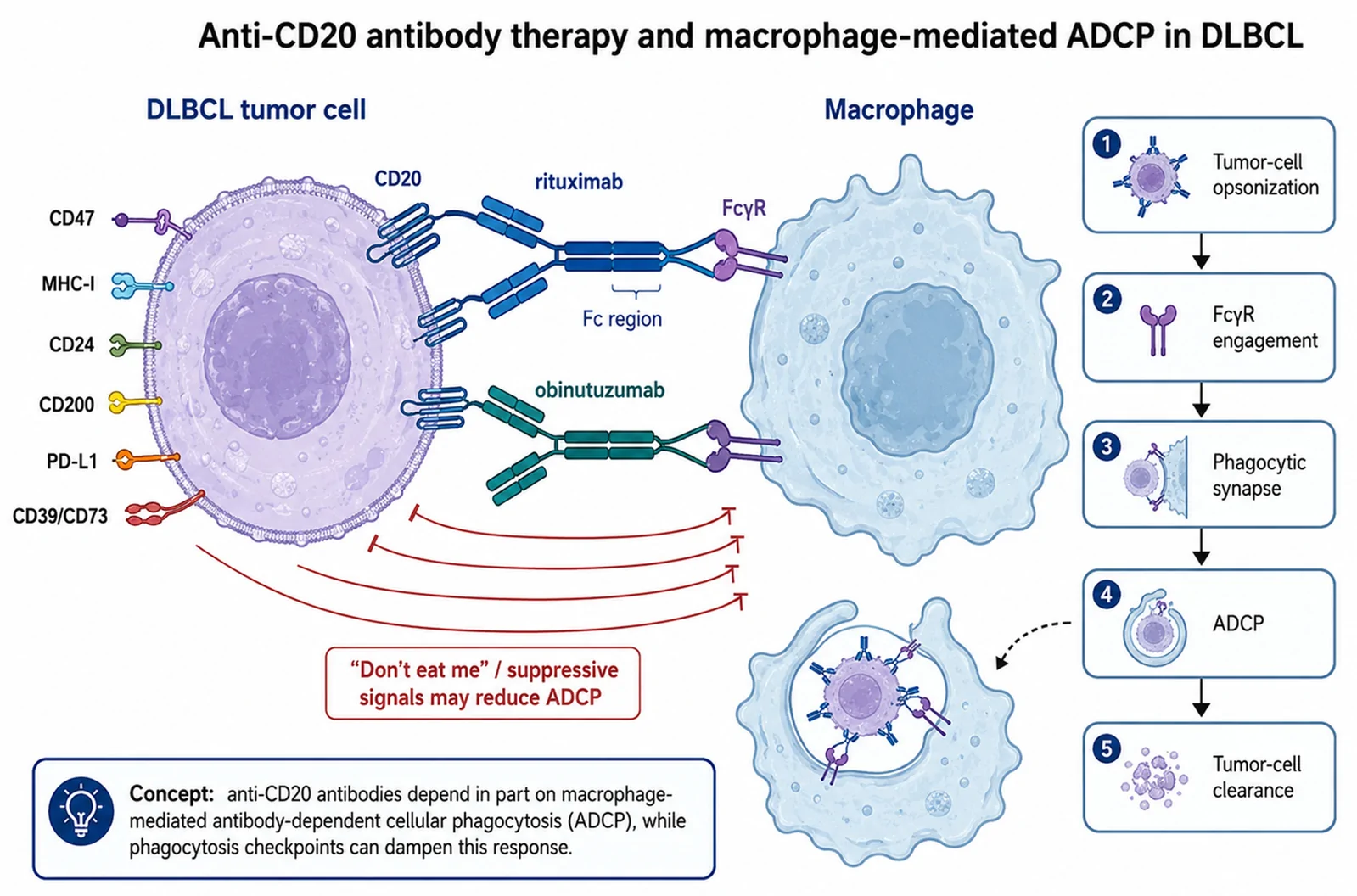
Antibody-opsonized DLBCL cells and macrophage-mediated ADCP. Rituximab or obinutuzumab binds CD20 on the lymphoma cell, while the Fc region engages Fc gamma receptors on macrophages. The numbered steps show tumor-cell opsonization, Fc receptor engagement, formation of a phagocytic synapse, ADCP, and tumor-cell clearance. Red inhibitory lines indicate signals that can interfere with productive phagocytosis. The figure is schematic and does not imply that all checkpoints are co-expressed in every DLBCL sample.

Anti-CD20 therapy provides the clearest setting in which to examine macrophage checkpoint biology. Rituximab or obinutuzumab first binds CD20 on the lymphoma cell, after which the antibody Fc region can engage Fc gamma receptors on macrophages. When that interaction is productive, a phagocytic synapse forms and the tumor cell can be internalized. **Figure 1** shows this sequence and where inhibitory tumor-macrophage signals may interfere with ADCP (25, 26, 28).

This biology is not confined to CD20. Tafasitamab is an Fc-engineered anti-CD19 antibody, and preclinical lymphoma studies have shown that blocking CD47/SIRPalpha can increase its macrophage-mediated antitumor activity (27). Tafasitamab is used clinically with lenalidomide in selected relapsed/refractory DLBCL, although the L-MIND regimen cannot separate macrophage effects from those of lenalidomide and other immune mechanisms (13). Even so, the anti-CD19 setting is useful because it asks whether a phagocytosis checkpoint remains relevant across more than one antibody platform.

The magnitude of ADCP depends on antigen density, antibody isotype and Fc engineering, macrophage state, Fc receptor repertoire, tumor-cell size and rigidity, and the balance between pro- and anti-phagocytic cues. DLBCL adds another layer of variability because cell-of-origin subtypes, stromal signatures, macrophage density, and checkpoint expression differ across cases (3–6, 20–22). A checkpoint axis that matters in one tissue context may therefore have little effect in another.

This heterogeneity argues for a network model rather than a single-marker model. Instead of asking whether CD200 alone explains anti-CD20 resistance, the framework asks a more rigorous and actionable question: which inhibitory axes are present, whether they are positioned to contact macrophages, and whether blocking them restores antibody-enhanced phagocytosis in a measurable assay.

## 5 Evidence-maturity map of macrophage phagocytosis checkpoint axes

**Figure 2:**
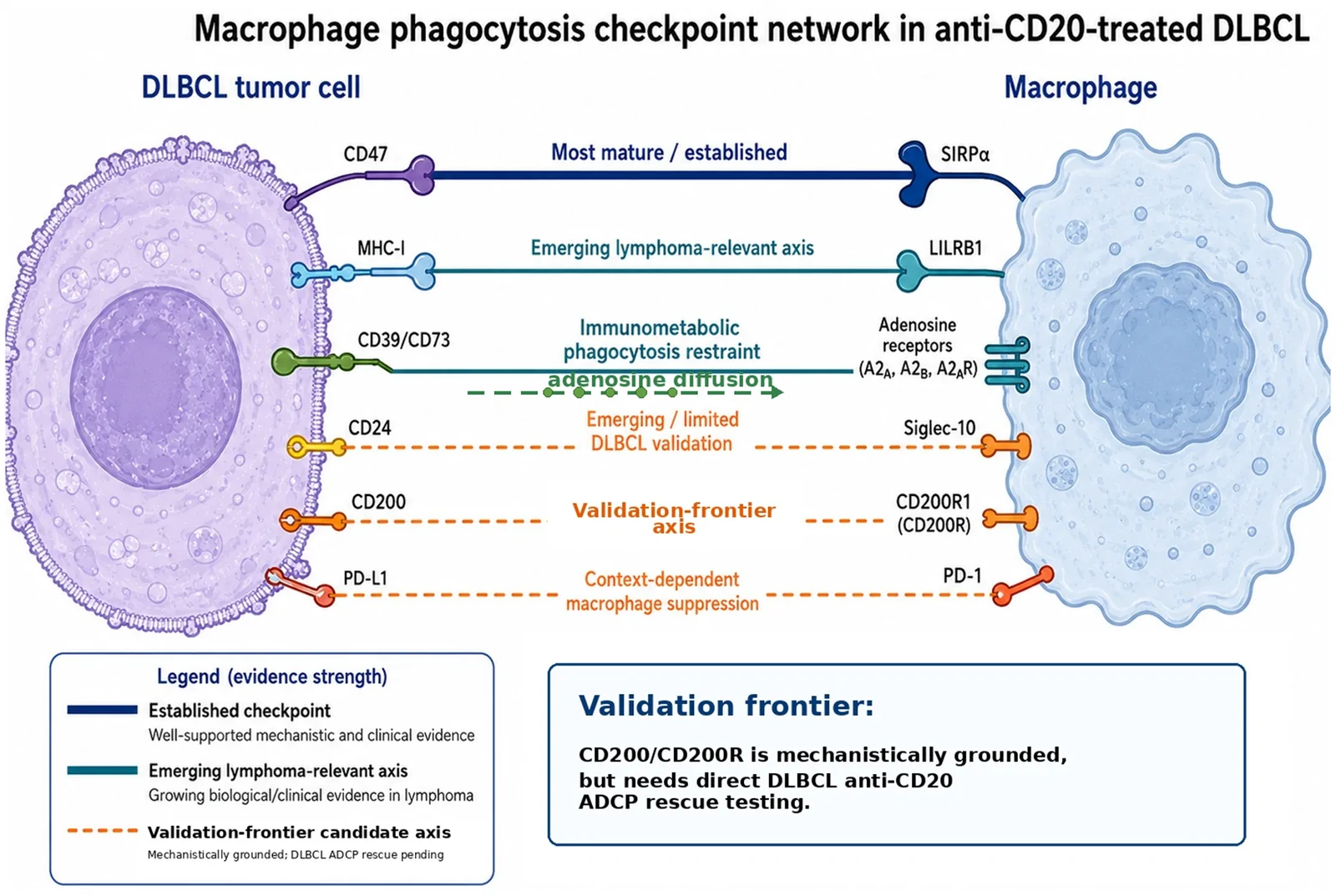
Evidence-maturity map of macrophage phagocytosis checkpoints in anti-CD20-treated DLBCL. Dark-blue connections indicate the CD47/SIRPalpha benchmark, teal connections indicate emerging lymphoma-relevant axes, and orange dashed connections indicate candidate or validation-frontier axes. CD39/CD73 is shown apart from the membrane checkpoints because adenosine acts through a diffusible metabolic pathway. CD200R1 in the schematic refers to CD200R. The diagram summarizes the relative maturity and mechanism of the evidence; it is not a quantitative comparison of pathway strength or expression.

**Figure 2** and **Table 3** give an overview of the checkpoint landscape. The axes differ in maturity, but they also differ in how they work. CD47/SIRPalpha, MHC-I/LILRB1, CD24/Siglec-10, and CD200/CD200R are contact-dependent membrane pathways. CD39/CD73 mainly acts through extracellular adenosine. That distinction matters when spatial assays and combination strategies are planned.

### 5.1 CD47/SIRPalpha: established benchmark

CD47/SIRPalpha is the reference checkpoint for this field. CD47 is broadly expressed on hematopoietic and malignant cells, while macrophage SIRPalpha provides an inhibitory signal that limits engulfment (30). In non-Hodgkin lymphoma models, CD47 blockade increased the effect of rituximab on macrophage phagocytosis and tumor clearance (25). Clinical studies later provided proof of concept for CD47-directed combinations, but they also exposed practical limitations, including anemia, antigen-sink effects, tolerability, and uneven development across programs (26, 31–34). We therefore use CD47/SIRPalpha as the Established benchmark in **Table 3**, while avoiding the implication that the pathway is already a settled DLBCL standard.

### 5.2 MHC-I/LILRB1: emerging co-checkpoint

MHC class I engagement of macrophage LILRB1 provides a second inhibitory signal that is not simply a duplicate of CD47/SIRPalpha (35). In lymphoma models, combined blockade of CD47 and LILRB1 increased CD20 antibody-dependent phagocytosis more than either pathway would be expected to explain alone (36). This gives LILRB1 direct lymphoma-relevant functional support, but the clinical and biomarker evidence remains limited. We therefore place MHC-I/LILRB1 in the Emerging tier (**Table 3**).

### 5.3 CD39/CD73-adenosine: emerging immunometabolic restraint

CD39/CD73-adenosine differs from the membrane checkpoints because its suppressive effect is mediated mainly through extracellular purine metabolism and adenosine receptor signaling. In B-cell lymphoma models, CD39 inhibition increased macrophage ADCP, and interrupting adenosine-mediated suppression improved anti-CD20 antitumor activity (37, 38). These data justify an Emerging designation, but future clinical work will need evidence that the adenosine pathway is actually inhibited in the tumor microenvironment rather than inferred from target expression alone (**Table 3**).

### 5.4 CD24/Siglec-10: candidate/emerging

The case for CD24/Siglec-10 in DLBCL is less complete. The axis has convincing pan-cancer checkpoint biology, and CD24 has been linked to an immune-cold phenotype in aggressive large B-cell lymphoma (39, 40). What is still missing is a direct DLBCL experiment showing that CD24/Siglec-10 blockade restores anti-CD20 ADCP. For that reason, we keep the axis in the Candidate/Emerging tier (**Table 3**).

### 5.5 CD200/CD200R: validation-frontier candidate/emerging and structural-functional bridge

CD200/CD200R needs a more cautious reading in DLBCL. In this review, CD200R is used as the shorthand for CD200 receptor 1 (CD200R1), except when the original article title or search term uses CD200R1. The biological rationale is clear: CD200 is an immunoregulatory ligand, CD200R is enriched on myeloid-lineage cells, and receptor engagement can suppress myeloid activation through Dok-family adaptors and RasGAP signaling (41–51). Recent work has also identified CD200-CD200R as part of the wider phagocytosis-checkpoint landscape (52). CD200 has also been linked to immune suppression or adverse biology across cancer contexts, including several hematologic malignancies such as acute myeloid leukemia, chronic lymphocytic leukemia, multiple myeloma, and B-cell lymphoma (53–58). These observations make the axis worth testing, but they do not yet establish a DLBCL therapeutic checkpoint.

The remaining DLBCL-specific evidence gap is narrow, important, and experimentally tractable. Protein-level evidence in large B-cell lymphoma is heterogeneous, with stronger and more consistent signals in PMBCL than in DLBCL-NOS (59–63). Most importantly, the decisive functional experiment is now clearly defined: CD200-high DLBCL cells, CD200R-positive macrophages, anti-CD20 antibody opsonization, and CD200/CD200R blockade should be tested together in a controlled ADCP assay with a measurable increase in phagocytic index. Public transcriptomic or immune-deconvolution observations cannot substitute for this experiment because they do not prove membrane CD200 protein, CD200R-positive macrophage proximity, receptor engagement, macrophage suppression, or restoration of anti-CD20 ADCP.

We therefore place CD200/CD200R in the Validation-frontier Candidate/Emerging tier rather than among established DLBCL checkpoints (**Table 3**). The missing experiment is straightforward to define: CD200-positive lymphoma cells and CD200R-positive macrophages should be shown to occupy the same contact-permissive niche, and pathway blockade should produce a reproducible increase in anti-CD20 ADCP. **Supplementary Figure S2** summarizes this proposed path from protein and spatial evidence to functional validation.

A deeper CD200/CD200R model requires connecting ligand-receptor recognition to macrophage function. Crystallographic work on the CD200/CD200R family, including the CD200-CD200R extracellular complex structure represented by PDB 4BFI, shows that both molecules contain Ig-like domains and interact through their N-terminal domains in a geometry compatible with immunological synapse-like cell-surface contact (64). This structural information does not replace human DLBCL co-culture evidence, but it strongly supports a contact-dependent checkpoint model in which tumor-cell CD200 can engage CD200R-positive macrophages when the two cell types occupy the same tissue neighborhood.

The intracellular mechanism of CD200R further strengthens the rationale for treating this axis as distinct from CD47/SIRPalpha. CD200R should not be described as a classical ITIM-SHP1/2 inhibitory receptor. Human CD200R-mediated inhibition of myeloid activation depends on a cytoplasmic NPLY/PTB-domain-binding motif that directly recruits Dok2 and subsequently engages RasGAP, distinguishing it from many inhibitory receptors that use ITIMs and SHP-family phosphatases (49). This matters for DLBCL because CD200R should not be treated as a simple weaker copy of SIRPalpha. Instead, CD200R-Dok2-RasGAP and SIRPalpha-SHP1/2 may converge on macrophage activation from different proximal signaling modules.

In anti-CD20-treated DLBCL, the key functional bridge is integration between Fc gamma receptor activating signals and CD200R inhibitory signaling in the same macrophage. Anti-CD20-opsonized lymphoma cells activate Fc gamma receptor-associated ITAM signaling, Syk-family kinases, PI3K and Ras/MAPK activity, actin remodeling, phagocytic synapse formation, and tumor-cell internalization. The central testable DLBCL hypothesis is that CD200R-Dok2-RasGAP signaling dampens this Fc receptor program at the level of Ras/MAPK-dependent inflammatory activation, cytoskeletal remodeling, or phagosome formation. Direct DLBCL phosphoproteomic, kinetic, and ADCP-rescue evidence now becomes the key missing layer rather than a reason to exclude the axis. Accordingly, quantified EC50 values, Ras-GTP hydrolysis rates, competitive phosphorylation behavior, and blockade-induced ADCP rescue should be treated as measurable endpoints for future macrophage-lymphoma co-culture studies.

PMBCL and PMBCL-like LBCL provide a particularly important validation context because CD200 immunostaining has diagnostic utility in PMBCL, whereas DLBCL-NOS shows more heterogeneous CD200 expression (60). In addition, 9p24.1 copy-number alterations involving CD274/PD-L1, PDCD1LG2/PD-L2, and JAK2 identify a subset of DLBCL with PMBCL-like features and high PD-L1/PD-L2/JAK2 expression (7). This raises a strong validation hypothesis of a dual inhibitory niche in which CD200/CD200R-mediated myeloid regulation coexists with PD-1 ligand-mediated T-cell suppression. The clinical implication is that PMBCL or PMBCL-like LBCL may be the most rational first setting for CD200 protein mapping, CD200R-positive macrophage proximity analysis, PD-L1/PD-L2 co-localization, and anti-CD20 ADCP rescue testing, without prematurely treating CD200 as an established PMBCL therapeutic target.

### 5.6 Macrophage PD-1/PD-L1: candidate/context

PD-1/PD-L1 is well established in T-cell checkpoint biology and has been clinically investigated in DLBCL (65, 66), but its role as a macrophage phagocytosis checkpoint is much less certain. Direct evidence that macrophage PD-1/PD-L1 signaling regulates anti-CD20 antibody-dependent cellular phagocytosis (ADCP) in DLBCL remains limited. Compared with CD47/SIRPalpha, MHC-I/LILRB1, and CD39/CD73-adenosine signaling, the macrophage-specific functional evidence is less developed. We therefore classify macrophage PD-1/PD-L1 as Candidate/context in **Table 3**.

### 5.7 Subtype-specific checkpoint hypotheses and validation priorities

Checkpoint relevance is unlikely to be uniform across DLBCL. Germinal center B-cell-like (GCB) DLBCL, activated B-cell-like (ABC)/non-GCB DLBCL, PMBCL, double-hit or high-grade LBCL, and relapsed or post-antibody disease can differ in ligand expression, macrophage density, antigen-presentation programs, cytokine tone, and treatment-induced myeloid remodeling. Because subtype-resolved functional evidence is still sparse for most axes, **Table 4** presents these differences as hypotheses to test rather than as fixed rules.

**Table 4:** Subtype-specific checkpoint hypotheses and validation priorities in DLBCL/LBCL.

DLBCL/LBCL context	Checkpoint hypothesis	Evidence status	Validation priority
GCB-DLBCL	CD47/SIRPalpha and MHC-I/LILRB1 may influence anti-CD20 ADCP, but subtype-specific functional evidence is limited.	Hypothesis-generating	IHC/flow ligand profiling, receptor-positive TAM density, spatial proximity, and ADCP rescue assays.
ABC/non-GCB DLBCL	Chronic inflammatory and NF-kappaB-rich contexts may increase relevance of CD39/CD73-adenosine and macrophage immune-state regulators.	Emerging hypothesis	CD39/CD73 protein, adenosine-pathway pharmacodynamics, macrophage-state mapping, and anti-CD20 ADCP testing.
PMBCL/PMBCL-like LBCL	CD200 expression and 9p24.1/PD-L1/PD-L2 programs may coexist, suggesting a dual myeloid and T-cell inhibitory niche.	Stronger CD200 protein rationale; DLBCL ADCP validation pending	CD200-CD200R-PD-L1/PD-L2 spatial co-localization, CD200R-positive macrophage mapping, and CD200/CD200R blockade ADCP rescue.
Double-hit/high-grade LBCL	Checkpoint landscape is insufficiently defined and should not be extrapolated from unselected DLBCL.	Evidence gap	Subtype-resolved multi-omic and functional validation.
Relapsed/refractory or post-antibody DLBCL	Therapy pressure may remodel TAMs and induce compensatory checkpoint co-expression.	Conceptual temporal model	Paired baseline/on-treatment biopsies, spatial profiling, Fc receptor competence, and ex vivo ADCP assays.

PMBCL and PMBCL-like LBCL are the strongest CD200-oriented validation context because CD200 has diagnostic utility in PMBCL, while 9p24.1-driven PD-L1/PD-L2 immune-evasion programs may coexist in PMBCL-like tumors (7, 60). ABC/non-GCB tumors may warrant stronger attention to inflammatory and adenosine-related programs, whereas GCB or double-hit/high-grade LBCL require cautious interpretation because checkpoint expression and macrophage proximity remain insufficiently mapped. Relapsed or post-antibody DLBCL should be treated as a separate biological state, because repeated immune pressure may remodel TAMs and induce compensatory checkpoint co-expression.

### 5.8 Cross-checkpoint relationships, pharmacology, and combination logic

These pathways should not be treated as interchangeable versions of the same ‘don’t eat me’ signal. CD47/SIRPalpha, MHC-I/LILRB1, CD24/Siglec-10, and CD200/CD200R all require cell-cell contact, but they use different proximal signaling mechanisms. CD39/CD73-adenosine works over a broader metabolic range. The strongest lymphoma evidence for non-redundancy comes from dual CD47/LILRB1 blockade, which increased CD20-dependent phagocytosis beyond single-axis inhibition (36). This supports testing combinations when two inhibitory pathways are present in the same tumor-macrophage niche, but expression alone is not enough to justify dual blockade.

Target biology also has to be considered alongside pharmacology. CD47 has strong functional evidence, but its broad expression on normal cells creates antigen-sink and anemia problems that influenced the development of priming-maintenance dosing strategies (26, 31). CD200/CD200R may be more context restricted, but it has much less DLBCL-specific functional evidence. The antibody platform matters as well. Obinutuzumab changes Fc receptor engagement through glycoengineering, while tafasitamab offers an anti-CD19 setting in which macrophage checkpoint blockade can also be tested (27, 28, 67).

A sensible combination strategy therefore needs more than two positive stains. At minimum, the ligand should be present on the tumor cell, receptor-positive macrophages should be nearby, Fc receptor competence should be preserved, and blockade should produce a measurable gain in ADCP. For metabolic pathways such as CD39/CD73, pharmacodynamic evidence of pathway inhibition is also important. When dual blockade is tested, quantitative interaction measures such as Bliss independence or Loewe additivity can help distinguish true cooperation from two unrelated single-agent effects.

## 6 Macrophage checkpoints in the modern DLBCL immunotherapy era

Macrophage checkpoints now have to be considered alongside anti-CD20 immunochemotherapy, polatuzumab-containing regimens, Fc-engineered anti-CD19 therapy, antibody-drug conjugates, CD20/CD3 bispecific antibodies, and CD19-directed CAR-T-cell therapy. **Table 2** summarizes where macrophages may contribute directly and where their role is more indirect. This treatment section is intentionally broader than the evidence map. It provides clinical context for the checkpoint framework but does not alter the evidence tier assigned to any pathway (8–16). B-cell-targeted macrophage-checkpoint constructs, including CD47/CD20 bispecific formats, offer a more direct way to combine tumor targeting with checkpoint disruption (68).

For anti-CD20 antibodies, macrophage ADCP is a direct effector mechanism and remains the cleanest setting for checkpoint testing. Anti-CD19 therapy provides a useful comparison. Tafasitamab has an Fc-engineered CD19-binding backbone, and CD47/SIRPalpha blockade increased its macrophage-mediated antitumor activity in preclinical lymphoma models (27). The clinical activity of tafasitamab plus lenalidomide should not be attributed to macrophages alone, but these data show that the checkpoint question is not limited to CD20-directed antibodies (13).

For bispecific antibodies and CAR-T-cell therapy, macrophage checkpoints may act more indirectly. Macrophages can shape antigen presentation, inflammatory tone, cytokine networks, myeloid suppression, and tissue-level immune architecture. A macrophage-suppressive microenvironment may limit productive T-cell engagement even when the primary therapeutic mechanism is T-cell redirection or engineered T-cell cytotoxicity. Conversely, treatment-induced tumor-cell death may create opportunities for macrophage-mediated clearance and antigen spreading. These considerations suggest that macrophage checkpoint profiling may eventually help refine combination strategies, but such applications require biomarker-enriched and mechanism-linked validation rather than broad pathway extrapolation.

## 7 Validation roadmap and biomarker-enriched trial design

**Figure 3:**
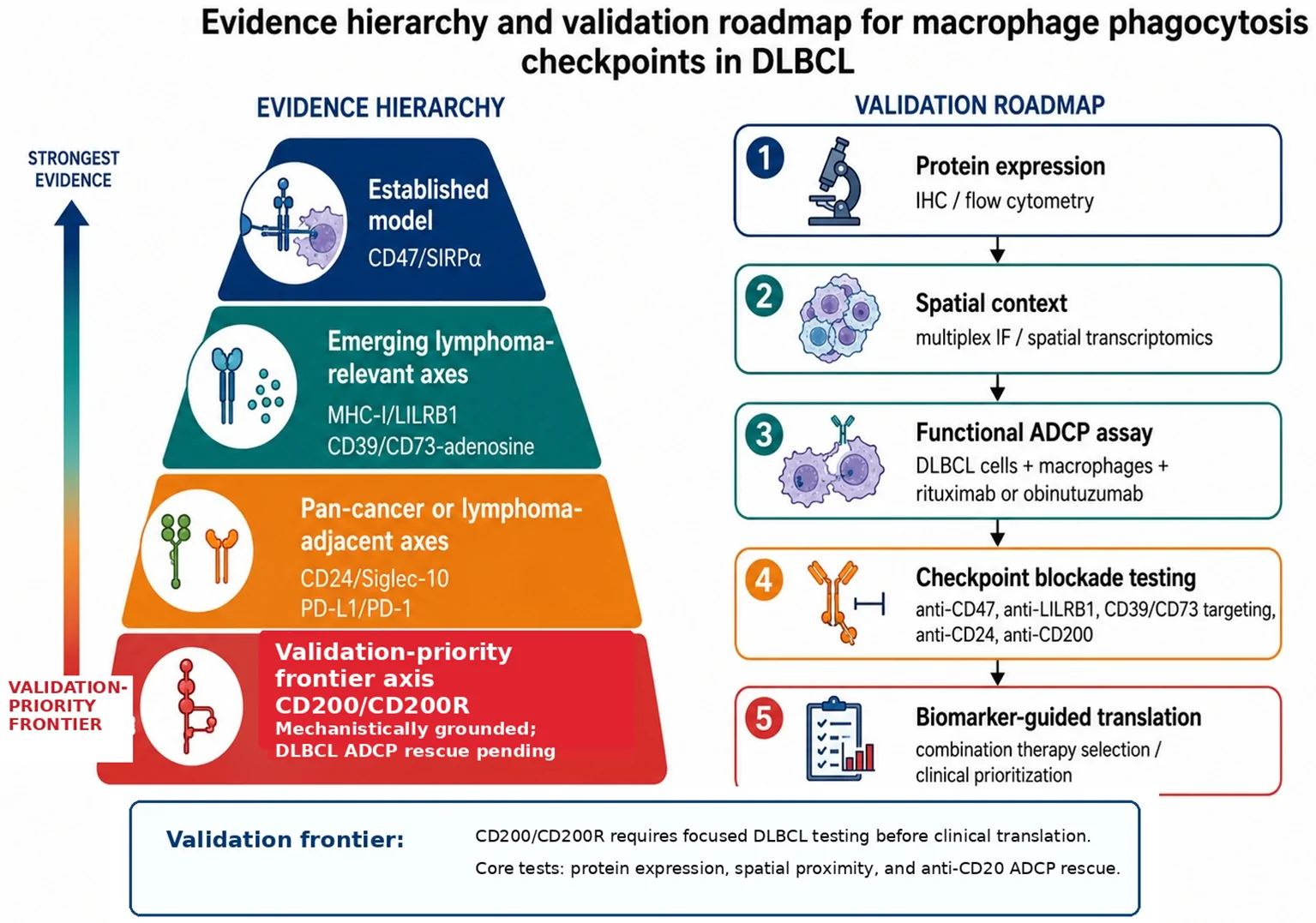
Evidence hierarchy and validation roadmap for macrophage phagocytosis checkpoints in DLBCL. The left panel groups the major axes by current evidence maturity. The right panel shows the proposed progression from protein expression and spatial context to functional ADCP rescue, blockade studies, and biomarker-guided clinical testing. The hierarchy is qualitative and should not be read as a validated clinical score.

The next step is to test these candidates in a sequence that can distinguish target presence from target function. **Figure 3** gives the overall scheme and **Table 5** lists practical assays. The first step is protein expression. Tumor-cell ligands should be measured by flow cytometry or immunohistochemistry (IHC), with multiplex immunofluorescence (IF) added when cell identity and tissue context matter. Transcript abundance alone does not establish that a target is present on the cell surface (69–76).

**Table 5:** Practical validation roadmap for candidate macrophage phagocytosis checkpoints in DLBCL.

Validation level	Suggested method	Surrogate endpoint	Trial design implication
Protein expression	Flow cytometry, immunohistochemistry (IHC), and multiplex immunofluorescence (IF) using prespecified cutoffs for CD47, CD200, CD24, CD39/CD73, MHC-I context, and PD-L1.	Screen-positive rate; reproducible ligand expression; assay concordance.	Defines eligibility and prevents unselected checkpoint trials.
Receptor-bearing macrophages	Multiplex IF, flow cytometry, single-cell RNA-seq with protein validation for SIRPalpha, LILRB1, CD200R, Siglec-10, and adenosine receptors.	Macrophage receptor density; TAM phenotype; Fc receptor profile.	Selects tumors capable of receiving inhibitory signals and responding to blockade.
Spatial proximity	Multiplex IF, imaging mass cytometry, or spatial transcriptomics to assess tumor-macrophage neighborhoods.	Tumor-macrophage contact score; spatial colocalization index.	Supports a cell-cell interaction mechanism and stratification variable.
Functional ADCP	Macrophage plus DLBCL co-culture with rituximab/obinutuzumab/tafasitamab and checkpoint blockade; include Fc controls.	Phagocytic index; tumor-cell engulfment; Fc gamma receptor activation.	Serves as pharmacodynamic endpoint and prioritizes combinations before patient exposure.
Combination logic	Single versus dual blockade; Fc-silent controls; macrophage activation and toxicity assays.	Additive ADCP without excessive inflammatory activation or cytopenia signal.	Guides CD47 plus LILRB1, CD47 plus CD39/CD73, or CD47 plus candidate-axis strategies.
Clinical translation	Prospective phase Ib/II trial with mandatory baseline and on-treatment biospecimens.	ctDNA molecular response, PET/CT metabolic response, macrophage activation, ORR, duration of response, progression-free survival (PFS).	Connects the framework to patient selection, surrogate endpoints, and go/no-go criteria.
CD200 structural-functional bridge	Map CD200 surface protein, CD200R-positive macrophages, and CD200/CD200R ligand-receptor proximity; integrate with CD200R-Dok2-RasGAP signaling markers and anti-CD20 ADCP assays.	CD200-CD200R proximity score; phospho-signaling or downstream macrophage activation readouts; ADCP rescue index.	Moves CD200/CD200R from biological plausibility to mechanism-linked validation before any clinical extrapolation.
Temporal TAM remodeling	Longitudinal baseline, early on-treatment, and relapse biopsies; multiplex IF, flow cytometry, spatial profiling, and ex vivo ADCP at each state.	Change in TAM phenotype, checkpoint co-expression, Fc receptor competence, phagocytic index, ctDNA/PET response.	Separates baseline candidate expression from treatment-induced or relapse-associated suppressive remodeling.
Spatial checkpoint ecology	Tumor-macrophage contact zones and paracrine/metabolic neighborhoods assessed by multiplex IF, imaging mass cytometry (IMC), co-detection by indexing (CODEX/PhenoCycler), CosMx, or spatial transcriptomics.	Ligand-receptor co-localization, nearest-neighbor distance, cellular niche assignment.	Prevents ligand-only interpretation and identifies contact-dependent versus paracrine checkpoint candidates.
CPIE-based prioritization	CPIE = (L x R x S x A)/(C x T), using ligand expression, receptor-positive TAM density, spatial proximity, ADCP rescue, compensatory burden, and toxicity risk.	Relative CPIE category after calibration in translational cohorts.	Guides single-axis blockade, dual blockade, or biomarker-enriched validation-only designs.

The second step is spatial. Receptor-bearing macrophages need to be in the same tissue neighborhood as ligand-positive lymphoma cells if a contact-dependent checkpoint is to function. Multiplex IF, imaging mass cytometry (IMC), or spatial transcriptomics can quantify tumor-macrophage distance, receptor-positive TAM density, and contact-permissive neighborhoods. Single-cell transcriptomics can help assign the signal to malignant or immune cells, but it does not replace protein or spatial confirmation.

The third level is functional evidence. The most direct test is an ADCP assay using checkpoint-high DLBCL cells, macrophages, and rituximab or obinutuzumab. The design should include single-axis blockade, dual-axis blockade, and appropriate Fc controls. A convincing result would show a reproducible increase in phagocytic index, not only a change in cytokine expression or gene-set enrichment.

Clinical translation should then begin with biomarker-enriched rather than unselected cohorts. For CD200/CD200R, a reasonable entry population would be CD200-positive LBCL/DLBCL or PMBCL-like tumors with nearby CD200R-positive macrophages and ex vivo evidence that blockade improves anti-CD20 ADCP. Early endpoints could include target occupancy, change in ex vivo phagocytic index, macrophage activation in on-treatment biopsies, circulating tumor DNA (ctDNA) response, positron-emission tomography (PET) metabolic response, and early overall response rate (ORR).

### 7.1 Conceptual checkpoint-prioritization score for candidate axes

The conceptual Checkpoint Inhibition Efficacy Index (CPIE) in **Table 6** is included as a way to organize these measurements, not as a validated clinical score. CPIE = (L x R x S x A)/(C x T), where L is tumor-cell ligand expression, R receptor-positive TAM density, S spatial co-localization, A ADCP rescue after blockade, C compensatory checkpoint burden, and T predicted toxicity risk. Possible inputs include IHC H-score or flow-cytometry mean fluorescence intensity (MFI), receptor-positive cells/mm2, nearest-neighbor distance, the change in phagocytic index after blockade, co-expressed alternative checkpoints, and normal-cell or red blood cell (RBC)/platelet cross-reactivity.

**Table 6:** Conceptual checkpoint inhibition efficacy index (CPIE) for prioritizing macrophage-checkpoint validation.

Parameter	Meaning	Suggested measurement	Interpretation
L	Tumor-cell ligand abundance	IHC H-score or flow-cytometry MFI	Defines target availability on malignant B cells.
R	Receptor-positive TAM density	cells/mm2 by multiplex IF, IMC, CODEX, or flow cytometry	Defines whether a receptor-bearing effector compartment is present.
S	Spatial co-localization	Manders coefficient, nearest-neighbor distance, or tumor-macrophage contact score	Defines whether ligand and receptor are positioned to interact.
A	ADCP rescue index	Fold change in phagocytic index after blockade in anti-CD20-opsonized co-culture	Defines whether blockade restores functional phagocytosis.
C	Compensatory checkpoint burden	Number or intensity of co-expressed alternative checkpoints	High C favors dual- or multi-axis validation.
T	Predicted toxicity risk	Normal-cell expression, RBC/platelet cross-reactivity, antigen-sink burden	High T requires cautious dosing, tumor-directed formats, or exclusion from systemic blockade.

This score is proposed as a calibration-ready design framework rather than as a current clinical decision tool. Before calibration, a high relative CPIE would support functional validation of single-axis blockade; an intermediate CPIE with high compensatory burden would support dual-blockade testing such as CD47 plus LILRB1 or CD47 plus adenosine-pathway targeting; and a low CPIE or unvalidated ADCP-rescue component would restrict the axis to biomarker-enriched exploratory cohorts. CD47/SIRPalpha would be expected to score high for functional maturity but also high for toxicity risk, whereas CD200/CD200R would remain in the validation-frontier tier until the A component, namely anti-CD20 ADCP rescue after CD200/CD200R blockade, is demonstrated in DLBCL or PMBCL-like LBCL.

## 8 Public-resource observations as hypothesis-prioritization context

Public transcriptomic resources can help identify DLBCL or LBCL contexts in which candidate macrophage checkpoint ligands are more likely to be expressed and therefore most suitable for validation. However, such observations should remain at the earliest stage of hypothesis prioritization. Transcript abundance cannot establish surface protein expression, cell-cell proximity, receptor engagement, or functional suppression of antibody-mediated phagocytosis. For this reason, public-resource observations are not used in this article to upgrade evidence tiers, define therapeutic relevance, or support clinical biomarker claims. Instead, they are used only to motivate future validation strategies, such as selecting CD200-positive LBCL/DLBCL or PMBCL-like samples for protein, spatial, and ADCP assays.

## 9 Limitations

Several limitations are important. This is a structured narrative review, so it does not provide pooled effect estimates. The checkpoint search was supplemented by hand-searching, citation tracking, selected journal checks, and ClinicalTrials.gov, and some relevant studies may still have been missed. We also used a separate treatment-context search to describe current US and European DLBCL therapy. Those treatment records were not included in the checkpoint evidence-tier calculation, which keeps the two questions separate but means that Section 6 should not be read as a systematic treatment review. Trial registrations were used to describe development status rather than as evidence of efficacy.

DLBCL heterogeneity introduces further limitations. This review does not fully stratify checkpoint expression across GCB, ABC, double-hit/high-grade, PMBCL-like, primary refractory, relapsed/refractory, and treatment-sensitive DLBCL states. Pediatric, adolescent/young-adult, very elderly, and frail populations are also underrepresented in the available evidence, and their immune microenvironments may differ from those in standard adult cohorts.

Exploratory public-resource observations were deliberately kept outside the evidence-grading framework. Transcript abundance is not the same as surface protein expression; bulk-tissue correlations cannot determine malignant-cell versus microenvironmental source; immune-deconvolution outputs vary by algorithm; available datasets often lack uniform treatment annotation, macrophage functional state, and on-treatment dynamic samples; and correlation cannot prove causality or ADCP rescue. These limitations define the proper role of public-resource signals: they support validation prioritization but do not replace the protein, spatial, and functional evidence needed to move CD200/CD200R from the validation frontier toward clinical translation.

## 10 Conclusions

Macrophage phagocytosis checkpoints help explain why an antibody-opsonized lymphoma cell may still escape clearance. The evidence, however, is uneven across pathways. CD47/SIRPalpha remains the Established benchmark. MHC-I/LILRB1 and CD39/CD73-adenosine are Emerging, CD24/Siglec-10 remains Candidate/Emerging, and macrophage PD-1/PD-L1 is better treated as Candidate/context. CD200/CD200R is notable because the mechanistic case is already fairly coherent, while the decisive DLBCL anti-CD20 ADCP-rescue experiment has not yet been reported.

For now, CD200/CD200R belongs at the validation frontier rather than in the list of established therapeutic targets. The next steps are concrete: confirm tumor-cell CD200 and macrophage CD200R protein, show that the two cell types are close enough to interact, measure pathway-linked macrophage signaling, and then test whether blockade improves antibody-dependent phagocytosis. Public transcriptomic data may help identify samples worth studying, but they cannot replace these experiments.

This evidence-maturity framework is intended to prevent both under-recognition and overinterpretation of candidate macrophage checkpoints while preserving a clear translational direction. Its practical value lies in ranking the burden of proof for each axis and converting prioritized immune biology into testable protein-level, spatial, functional, and biomarker-enriched clinical validation strategies. By adding a structural-functional CD200/CD200R bridge, a spatial macrophage-checkpoint ecology, subtype-specific validation hypotheses, quantitative pharmacology, a conceptual CPIE score, and a temporal model of TAM remodeling, this framework advances a mechanism-linked validation agenda rather than a descriptive target list.

## Supplementary material

Supplementary Figure 1 Search strategy and working evidence-map selection record. Records from PubMed/MEDLINE, Web of Science Core Collection, targeted Google Scholar searching, and ClinicalTrials.gov were manually de-duplicated and screened for relevance to lymphoma, macrophage biology, ADCP, and phagocytosis checkpoints. This figure records the search process used for the structured narrative review; it is not presented as a PRISMA flow diagram. Exploratory public-transcriptomic observations were not used for evidence grading.

Supplementary Figure 2 CD200/CD200R validation-frontier hypothesis. The figure separates published pan-cancer CD200/CD200R phagocytosis biology from the DLBCL-specific evidence that is still missing. Moving the axis beyond the validation-frontier tier would require confirmation of tumor-cell CD200 protein, nearby CD200R-positive macrophages, and reproducible improvement of anti-CD20 ADCP after pathway blockade.

Supplementary Data Sheet 1 Full search strategy, screening record, evidence-grading rules, and the distinction between checkpoint evidence mapping and treatment-context searches. The data sheet contains the Web of Science search strings, PubMed/MEDLINE and supplementary searches, eligibility criteria, data-extraction fields, evidence-tier definitions, and the rules used for exploratory public-resource observations.

## Glossary

ABC: activated B-cell-like
ADCC: antibody-dependent cellular cytotoxicity
ADCP: antibody-dependent cellular phagocytosis
CAR: chimeric antigen receptor
CPIE: Checkpoint Inhibition Efficacy Index
ctDNA: circulating tumor DNA
DLBCL: diffuse large B-cell lymphoma
FcγR: Fc gamma receptor
GCB: germinal center B-cell-like
IHC: immunohistochemistry
IF: immunofluorescence
IMC: imaging mass cytometry
LBCL: large B-cell lymphoma
LILRB1: leukocyte immunoglobulin-like receptor B1
MFI: mean fluorescence intensity
MHC-I: major histocompatibility complex class I
NHL: non-Hodgkin lymphoma
ORR: overall response rate
PD-1: programmed cell death protein 1
PD-L1: programmed death-ligand 1
PET: positron-emission tomography
PFS: progression-free survival
PMBCL: primary mediastinal large B-cell lymphoma
RBC: red blood cell
SIRPalpha: signal regulatory protein alpha
TAM: tumor-associated macrophage
TLS: tertiary lymphoid structure
TME: tumor microenvironment.
