# Supplementary material for: Macrophage phagocytosis checkpoints in DLBCL immunotherapy: an evidence-maturity framework from CD47 to CD200
Source: Front Oncol. 2026 Sep 11;16:1933140. doi: 10.3389/fonc.2026.1933140 (PMC13613202; doi:10.3389/fonc.2026.1933140)
Supplement: Supplementary file 1 [file Table1.docx]

# Supplementary Data Sheet 1. Full search strategy, screening record, and evidence-grading rules

This supplementary data sheet records the structured checkpoint search, screening process, Web of Science Core Collection search strings, PubMed/MEDLINE and supplementary searches, evidence-grading rules, and the separate treatment-context checking used for the contemporary DLBCL treatment section.

## S1. Search sources and record counts

| Source | Search scope / terms | Records identified | Role in evidence map |
| --- | --- | --- | --- |
| PubMed/MEDLINE | DLBCL/large B-cell lymphoma/B-cell lymphoma AND anti-CD20/ADCP/macrophage AND CD47, LILRB1, CD39/CD73, CD24, CD200, PD-1/PD-L1 or related terms. | 252 | Main peer-reviewed biomedical literature source. |
| Web of Science Core Collection | Five Topic-field searches covering DLBCL, macrophage-mediated phagocytosis, anti-CD20 ADCP, CD47/SIRPalpha, CD200/CD200R, and candidate myeloid/phagocytosis-checkpoint axes. | 77; 133; 42; 213; 8 | Structured database search performed to strengthen methodological reproducibility. |
| Google Scholar targeted hand-searching | Five targeted queries covering CD47/SIRPalpha, LILRB1, CD200/CD200R, CD24/Siglec-10, and CD39/CD73 in lymphoma phagocytosis contexts; first one to two result pages screened; citation chasing from anchor papers performed. | 18 candidate records | Supplementary source for recent mechanistic papers and citation-chain checking. |
| ClinicalTrials.gov | magrolimab lymphoma rituximab; evorpacept lymphoma; CD47 lymphoma; SIRPalpha lymphoma. | 42 records before de-duplication | Mapping of clinical development activity; not used as efficacy proof. |
| De-duplication and screening | Manual de-duplication across sources and title/abstract screening for lymphoma, macrophage, ADCP, and checkpoint relevance. | 33 included in working evidence map | Produced the evidence map used for the present draft. |
| High-impact lymphoma/cancer-immunology journal checking | Targeted checking of recent issues and article records from lymphoma, hematology, and cancer-immunology journals for macrophage checkpoint, anti-CD20, and ADCP terms. | Not counted separately | Risk-mitigation and update-checking step; no independent efficacy inference was drawn from this step. |
| Contemporary DLBCL treatment-context checking | Current US and European LBCL guidance, plus targeted checks of anti-CD19 antibodies, antibody-drug conjugates, CD20/CD3 bispecific antibodies, and CAR-T therapy. | Not counted separately | Used only to provide context for the modern-treatment section and Table 3; not included in checkpoint evidence-tier assignment. |

## S2. Web of Science Core Collection Topic-field searches

| Search ID | Search theme | Field | Records identified |
| --- | --- | --- | --- |
| WOS-1 | DLBCL, macrophage/phagocytosis, and immune checkpoint | Topic | 77 |
| WOS-2 | DLBCL, anti-CD20 antibodies, and ADCP/macrophage phagocytosis | Topic | 133 |
| WOS-3 | DLBCL and CD47/SIRPalpha macrophage checkpoint | Topic | 42 |
| WOS-4 | CD200/CD200R and macrophage/phagocytosis-related checkpoint biology | Topic | 213 |
| WOS-5 | Other candidate myeloid/phagocytosis checkpoints in DLBCL | Topic | 8 |

## S3. Full Web of Science Core Collection search strings

WOS-1. General DLBCL, macrophage/phagocytosis, and immune-checkpoint biology

("diffuse large B-cell lymphoma" OR DLBCL) AND (macrophage* OR "tumor-associated macrophage*" OR TAM OR myeloid OR phagocytosis OR efferocytosis OR ADCP OR "antibody-dependent cellular phagocytosis") AND ("immune checkpoint*" OR "phagocytosis checkpoint*" OR "eat me" OR "myeloid checkpoint*")

WOS-2. Anti-CD20 ADCP and macrophage-mediated phagocytosis

("diffuse large B-cell lymphoma" OR DLBCL) AND (rituximab OR obinutuzumab OR "anti-CD20" OR "CD20 antibody" OR "CD20 antibodies") AND (phagocytosis OR ADCP OR macrophage* OR "antibody-dependent cellular phagocytosis")

WOS-3. CD47/SIRPalpha axis

("diffuse large B-cell lymphoma" OR DLBCL) AND (CD47 OR SIRPA OR "SIRP alpha" OR "SIRPalpha" OR "signal regulatory protein alpha") AND (macrophage* OR phagocytosis OR ADCP OR "anti-CD20" OR rituximab OR obinutuzumab)

WOS-4. CD200/CD200R axis; intentionally broadened to lymphoma and cancer terms to capture pan-cancer mechanistic phagocytosis-checkpoint evidence

(CD200 OR CD200R OR CD200R1) AND (macrophage* OR phagocytosis OR efferocytosis OR ADCP OR "immune checkpoint*" OR "myeloid checkpoint*" OR "phagocytosis checkpoint*") AND (lymphoma OR "diffuse large B-cell lymphoma" OR DLBCL OR cancer OR tumor OR tumour)

WOS-5. Candidate myeloid/phagocytosis-checkpoint axes in DLBCL; PD-1/PD-L1 and broad lymphoma terms removed to reduce T-cell-checkpoint noise

("diffuse large B-cell lymphoma" OR DLBCL) AND (LILRB1 OR LILRB2 OR ILT2 OR ILT4 OR SIGLEC10 OR SIGLEC15 OR CD24 OR "CD24-Siglec10" OR "MHC-I" OR "MHC class I" OR SLAMF7) AND (macrophage* OR phagocytosis OR ADCP OR "myeloid checkpoint*" OR "phagocytosis checkpoint*")

## S4. Google Scholar and ClinicalTrials.gov supplementary searches

| Source | Search term or strategy | Screening approach |
| --- | --- | --- |
| Google Scholar | CD47 SIRPalpha rituximab lymphoma phagocytosis | First one to two result pages screened; citation tracking from anchor papers. |
| Google Scholar | LILRB1 CD47 CD20 antibody lymphoma phagocytosis | First one to two result pages screened; citation tracking from anchor papers. |
| Google Scholar | CD200 CD200R phagocytosis checkpoint lymphoma | First one to two result pages screened; used only to identify relevant published mechanistic or lymphoma-adjacent evidence. |
| Google Scholar | CD24 Siglec-10 lymphoma macrophage phagocytosis | First one to two result pages screened. |
| Google Scholar | CD39 CD73 rituximab lymphoma macrophage phagocytosis | First one to two result pages screened. |
| ClinicalTrials.gov | magrolimab lymphoma rituximab; evorpacept lymphoma; CD47 lymphoma; SIRPalpha lymphoma | Trial records used to map clinical-development status, not to infer efficacy. |
| Guideline/review checking | 2025 EHA LBCL guideline, 2026 DLBCL management update, and recent first-line and relapsed/refractory treatment literature. | Used to describe current US and European treatment practice; not used to assign checkpoint evidence tiers. |
| Targeted therapy-context checking | Tafasitamab/CD19 DLBCL, polatuzumab DLBCL, glofitamab or epcoritamab DLBCL, and CAR-T therapy in large B-cell lymphoma. | Used to support the treatment-context table and to clarify why anti-CD19 therapy is discussed alongside the anti-CD20 literature. |

## S5. Eligibility criteria and exclusion categories

| Category | Criteria |
| --- | --- |
| Inclusion criteria | Peer-reviewed primary mechanistic, translational, biomarker, or clinical-development studies relevant to macrophage-mediated phagocytosis, ADCP, anti-CD20 antibody activity, or myeloid/phagocytosis-checkpoint axes in DLBCL, LBCL, B-cell lymphoma, or directly relevant pan-cancer contexts. |
| Contextual inclusion | Reviews were used to contextualize the field but were not counted as strong axis-level evidence unless they contained extractable primary information relevant to the evidence map. |
| Exclusion criteria | Records unrelated to lymphoma, macrophage biology, antibody therapy, phagocytosis, or immune-checkpoint mechanisms; non-biomedical records; duplicate records; and records without extractable evidence relevant to the evidence map. |
| ClinicalTrials.gov use | Trial records were used to map clinical development and maturity, not as proof of efficacy unless peer-reviewed clinical results were available. |
| CD200/CD200R boundary | Pan-cancer CD200/CD200R mechanistic evidence was used to inform biological plausibility but did not establish DLBCL-specific validation or anti-CD20 ADCP rescue. |

## S6. Data-extraction fields

- checkpoint axis and ligand-receptor pair
- disease context and DLBCL/LBCL specificity
- sample type or experimental model
- protein-level evidence in lymphoma or related B-cell neoplasms
- functional evidence for macrophage phagocytosis or antibody-mediated ADCP
- anti-CD20 context including rituximab or obinutuzumab when available
- in vivo lymphoma evidence
- clinical-trial or clinical-development evidence
- biomarker, predictive, or enrichment relevance
- negative, terminated, or inconclusive evidence
- main limitation affecting evidence tier assignment

## S7. Evidence-grading rules

| Tier | Definition |
| --- | --- |
| Established | Direct, peer-reviewed evidence in DLBCL, LBCL, or B-cell NHL, preferably replicated or supported by both mechanistic and translational data. Overall Established status required at least three established dimensions, including functional ADCP or clinical lymphoma evidence. |
| Emerging | At least one relevant primary lymphoma study or strong pan-cancer mechanistic evidence with lymphoma-adjacent support, but without independent validation or full clinical linkage. |
| Candidate/Emerging | Boundary tier used when a pathway has meaningful mechanistic plausibility or lymphoma-adjacent support but lacks direct DLBCL anti-CD20 ADCP rescue or sufficient clinical validation. |
| Candidate | Indirect data, non-lymphoma models, public-resource associations, review-level rationale, or mechanistic plausibility without direct DLBCL/LBCL functional validation. |
| Downgrading considerations | Negative, terminated, or inconclusive evidence could downgrade interpretation but was not used to erase valid mechanistic evidence. |

## S8. Boundary statement for exploratory public-transcriptomic observations

Exploratory public-transcriptomic analyses were not part of the literature search strategy and were not incorporated into the evidence-level assignment in Table 2. Such observations, when mentioned, were used only for hypothesis generation and sample-prioritization. Transcript-level associations cannot establish membrane protein abundance, tumor-macrophage spatial contact, receptor engagement, macrophage functional state, therapeutic actionability, or rescue of anti-CD20 ADCP. This boundary is particularly important for CD200/CD200R, where public deconvolution or correlation results should not be interpreted as evidence for macrophage-mediated immune suppression in DLBCL.

Nomenclature note: CD200R is used in the narrative as the shorthand for CD200 receptor 1. CD200R1 is retained where it appears in an original article title, formal gene/protein nomenclature, or a search synonym. Treatment-context searches were kept separate from the checkpoint evidence-tier calculation.
